# Dissecting and validation the biomarker of heart failure progression in patients with atherosclerosis by single-cell sequencing, bioinformatics, and machine learning

**DOI:** 10.3389/fgene.2025.1587274

**Published:** 2025-09-12

**Authors:** Lihua Ni, Huabo Li, Juan Du, Ke Zhou, Fugui Zhang, Liankai Wang

**Affiliations:** ^1^ Department of Nephrology, Zhongnan Hospital of Wuhan University, Wuhan, China; ^2^ Department of Cardiology, Minda Hospital of Hubei Minzu University, Enshi, China; ^3^ Hubei Provincial Key Laboratory of Occurrence and Intervention of Rheumatic Diseases, Minda Hospital of Hubei Minzu University, Enshi, China

**Keywords:** Heart failure progression, atherosclerosis, single-cell sequencing, bioinformatics, machine learning

## Abstract

**Objective:**

This study aimed to identify early biomarkers associated with the progression from atherosclerosis (AS) to heart failure (HF) by integrating single-cell RNA sequencing (scRNA-seq) and bulk transcriptomic data, and to explore the potential underlying mechanisms.

**Method:**

Transcriptomic datasets (GSE28829 and GSE57345) were obtained from the Gene Expression Omnibus (GEO) database, and single-cell RNA sequencing (scRNA-seq) data were downloaded from the Human Cell Landscape (HCL) platform. Genes of interest were identified by integrating results from weighted gene co-expression network analysis (WGCNA), differentially expressed genes (DEGs) analysis, and cell-type-specific expression patterns. Three machine learning algorithms (LASSO, Random Forest, and SVM-RFE) were employed to screen for robust candidate biomarkers. External validation was performed using three independent datasets: GSE53274, GSE5406, and GSE59867.

**Result:**

ScRNA-seq data screened for 2828 cardiac-related genes. WGCNA identified 918 genes highly associated with AS. In addition, the limma package identified 9675 DEGs associated with HF progression. A total of 119 overlapping genes were obtained by intersecting the results from the above three analyses. Based on these 119 overlapping genes, three machine learning algorithms (LASSO, Random Forest, and SVM-RFE) were applied to datasets GSE28829 and GSE57345, and consistently identified CD48 as a robust signature gene, with an area under the curve (AUC) greater than 0.7. External validation confirmed CD48 as a potential biomarker for the progression from AS to HF.

**Conclusion:**

CD48 was identified as a potential early biomarker for the transition from AS to HF, which may offer new insights for risk stratification and early intervention in disease progression.

## 1 Introduction

Atherosclerosis (AS) is a chronic, progressive disease characterized by low-grade inflammation of the arterial wall. Its core mechanism involves the subendothelial deposition of plasma apolipoprotein B (apoB)-containing lipids, which triggers immune-inflammatory responses and leads to the gradual formation of atherosclerotic plaques. These plaques can cause luminal narrowing or even occlusion of the affected arteries (Bäck et al., 2019). Once diagnosed, patients typically require long-term lipid-lowering and anticoagulant therapy, resulting in a significant reduction in quality of life.

More critically, AS serves as a fundamental pathological basis for a wide range of cardiovascular diseases. The rupture of unstable plaques and the subsequent formation of acute thrombi are central mechanisms in acute coronary syndromes (ACS), ischemic stroke, and peripheral arterial disease (Schaar et al., 2004; Brown et al., 2017; Fan et al., 2019). Among them, coronary artery disease (CAD) is a major cause of heart failure (HF). Studies indicate that approximately 36% of patients with acute myocardial infarction (AMI) develop chronic HF within 7–8 years following the initial event (Mosterd and Hoes, 2007), highlighting the importance of the AS → CAD → HF progression pathway. Additionally, shared risk factors such as smoking, diabetes, and obesity may exacerbate endothelial dysfunction and further accelerate the progression from CAD to HF (Ng et al., 2014). Although current treatments—including statins and PCSK9 inhibitors—have improved outcomes, substantial residual risk remains, particularly due to the lack of early biomarkers for identifying patients at risk of progression from AS to HF.

A variety of biomarkers have been used for the diagnosis and risk stratification of atherosclerosis (AS) and heart failure (HF). For example, lipoprotein-associated phospholipase A2 (Lp-PLA2) (Panta et al., 2022), high-sensitivity C-reactive protein (hs-CRP) (Ridker, 2016), and imaging-based markers (e.g., coronary calcium scoring) (Khan et al., 2023) have been widely applied in the assessment of AS. For HF, N-terminal pro-brain natriuretic peptide (NT-proBNP) is regarded as the gold-standard biomarker for diagnosis (Leto et al., 2016), while soluble suppression of tumorigenicity-2 (sST2) has been shown to help predict adverse outcomes (Mueller and Dieplinger, 2016). However, these biomarkers are generally disease-specific, targeting either AS or HF, and fail to capture the dynamic molecular changes occurring during the transition from AS to HF. For instance, although Lp-PLA2 effectively reflects AS burden, it has limited value in predicting HF risk; conversely, NT-proBNP is highly specific for HF diagnosis but cannot predict the progression to HF in AS patients.

In this study, we constructed a molecular landscape covering the progression from AS to HF using publicly available data from the Human Cell Landscape (HCL) platform and the Gene Expression Omnibus (GEO) database. Differentially expressed genes (DEGs) were identified between HF samples and healthy controls, and key genes highly correlated with AS were screened using weighted gene co-expression network analysis (WGCNA). We then intersected the cardiac-related genes obtained from single-cell sequencing (SCS), the AS-related genes from WGCNA, and the DEGs associated with HF. Subsequently, machine learning algorithms were applied to identify potential early biomarkers that may predict the progression from AS to HF.

## 2 Methods

### 2.1 Study design

**FIGURE 1:**
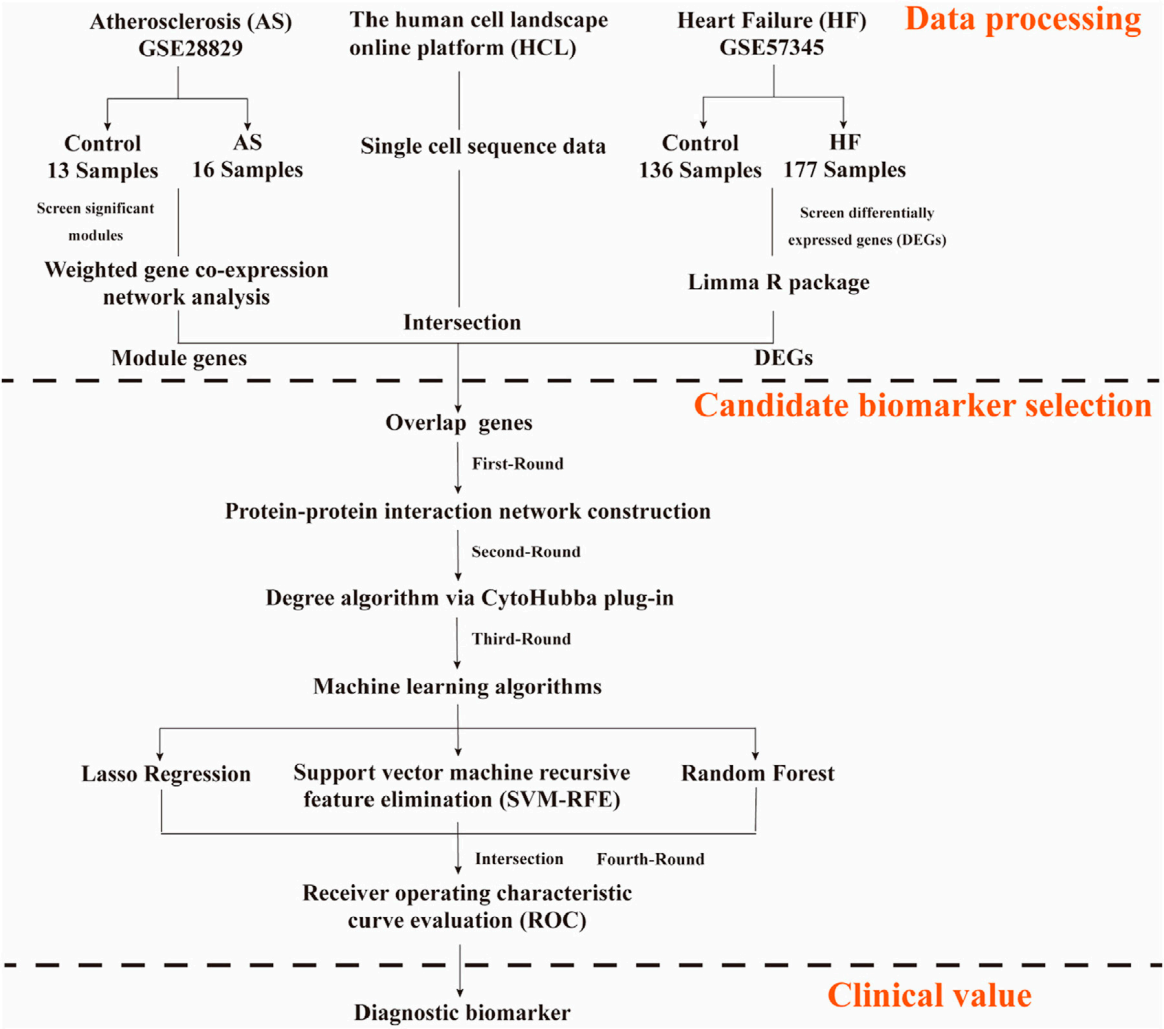
The flowchart of the study design and data processing.

By integrating differential expression analysis, WGCNA, single-cell transcriptomic data, and multiple machine learning approaches, this study aimed to systematically explore the key molecular mechanisms involved in the transition from atherosclerosis to heart failure. Figure 1 shows the flowchart of the study design and data processing. Datasets were retrieved from the GEO database (https://www.ncbi.nlm.nih.gov/geo/).

Gene expression profiling data GSE28829 (platform: GPL570 [HG-U133_Plus_2] Affymetrix Human Genome U133 Plus 2.0 Array) and GSE57345 (platform: GPL11532 [HuGene-1_1-st] Affymetrix Human Gene 1.1 ST Array [transcript (gene) version]) were obtained using the GEOquery package. After extracting the raw data, the background was calibrated, normalized, and log-transformed using the affy package (v 4.1.2). The limma package identifies DEGs in the GSE57345 dataset, with the filtering criteria of P < 0.05 and |Log (Fold change)| > 1. Genes with Log(Fold change) > 1 and p-value <0.05 were categorized as upregulated genes, while genes with Log(Fold change) < −1 and p-value <0.05 were categorized as downregulated genes, and we use volcano plots and venn diagrams to represent differentially expressed genes. The GSE28829 dataset includes transcriptomic data of human arterial tissues representing the progression of atherosclerotic lesions from early to advanced stages, making it suitable for constructing a temporal expression profile of AS progression (Döring et al., 2012). GSE57345, one of the largest expression datasets of adult myocardial tissues in heart failure to date, provides time-series data capturing the transition from compensatory hypertrophy to HF, thereby facilitating the identification of myocardial molecular features during the AS-HF progression (Liu et al., 2015). The combination of these two datasets fills the knowledge gap from vascular lesions to cardiac remodeling, representing two critical stages—AS and HF—and thereby supports the identification of cross-stage biomarkers in this study. For external validation, we have chosen GSE53274 (platform: GPL570 [HG-U133_Plus_2] Affymetrix Human Genome U133 Plus 2.0 Array) and GSE5406 (GPL96 [HG-U133A] Affymetrix Human Genome U133A Array). Since blood samples are easier to obtain, we also adopted the whole blood dataset (GSE59867) for external validation. Due to different platforms, batch correction is used for distinct datasets. The detailed informations of the datasets are shown in Table 1.

**TABLE 1:** The clinical information of included GEO datasets.

GEO dataset	Sample type	Sample size				Role	Platform
		Disease group		Control group			
GSE28829	Atherosclerotic carotid artery segments	Advanced (thin or thick fibrous cap atheroma) lesions	16	Early ((pathological) intimal thickening and intimal xanthoma)	13	Test dataset	GPL 6244
GSE57345	Cardiac tissue	ISCH patients and DCM patients	177	Individuals with normal hearts	136	Test dataset	GPL 17077
GSE53274	Atherosclerotic lesions	Restenotic lesion	5	Primary lesion	4	Validation dataset	GPL 570
GSE5406	Human myocardium tissue	Patients undergoing advanced systolic heart failure	196	Nonfailing controls	16	Validation dataset	GPL 96
GSE59867	Peripheral blood	STEMI patients progress into HF	32	STEMI patients without HF	30	Validation dataset	GPL 6244

The integration of single-cell transcriptomic data from the Human Cell Landscape (HCL) enables the resolution of cell type-specific expression patterns within the cardiac microenvironment, thereby avoiding signal dilution caused by tissue heterogeneity. For the single-cell sequencing (SCS) data, Han et al. (2020) performed a platform (Human Cell Landscape) including single cell sequencing on each organ/tissue sample of the Chinese Han population, which provided valuable data for revealing the complex cell types in each organ/tissue. Human Cell Landscape (HCL) was used to investigate the expression profiles of candidate gene markers in human normal adult cardiac tissue from SCS results (Zhang et al., 2022). First, the high-dimensional data were nonlinearly downscaled using the T-SNE algorithm to separate different cell clusters; second, the candidate gene markers in each cell cluster were identified using the “FindAllMarkers” package, with the screening thresholds for candidate gene markers: P < 0.05; and third, the expression of candidate gene markers in each cell cluster was used as background.

### 2.2 Weighted gene co-expression network analysis

Weighted Gene Co-expression Network Analysis (WGCNA) is an unsupervised network modeling method that is well-suited for identifying gene functional modules associated with specific phenotypes in complex systemic diseases. First, the correlation coefficients between gene pairs were calculated by Pearson’s correlation coefficient using the “WGCNA” package. According to the principle of scale-free network, a soft threshold (β = 16) was selected to sequentially construct scale-free co-expression network, and the neighbor-joining matrix was transformed into topological overlap matrix (TOM). Then, average association hierarchical clustering was performed based on TOM - based dissimilarity, and genes with similar expression profiles were divided into modules with at least 60 genes in each module, and each module was assigned a corresponding color. A dynamic hybrid branch-cutting method was used to identify module eigengenes (ME) on the TOM-based dendrogram. The gene significance measure based on phenotypic traits was defined as the absolute value of the correlation between gene i and the phenotypic trait (T): GS_i_ = |cor (i,T)|. Modules with |GS_i_| > 0.5 were used as key gene modules in this study.

### 2.3 Enrichment analysis

Gene Ontology (GO), Kyoto Encyclopedia of Genes and Genomes (KEGG) were performed using the R package “clusterProfiler”. DO is an important annotation for translating molecular discoveries from high-throughput data to clinical relevance. Disease Ontology (DO) analysis was performed using the “DOSE” package. DOSE is an R package that provides semantic similarity calculations between DO terms and genes, enabling biologists to explore similarities between disease and gene function from a disease perspective. This enables biologists to validate disease correlations in biological experiments and discover disease associations from high-throughput biological data (Yu et al., 2015). The top 30 data in terms of qvalue were plotted as count bar charts and bubble plots of generatio.

### 2.4 Construction of protein-protein interation (PPI) networks

The STRING database was used to construct PPI networks for overlapping genes, which were visualized by Cytoscape software (https://cytoscape.org). Then, to discover important modules and genes, the PPI network was evaluated using the CytoHubba module in Cytoscape.

### 2.5 Machine learning strategies for screening biomarkers

Candidate biomarkers of HF progression in atherosclerotic patients were identified by three machine learning algorithms: random forest (RF), least absolute shrinkage and selection operator (LASSO) regression, and support vector machine-recursive feature elimination (SVM). The use of three machine learning models in this study enables cross-validation, thereby enhancing the robustness of biomarker selection. First, random forest (RF) analysis is a decision tree-based machine learning method that assesses the importance of variables mainly by evaluating the importance of each variable (Alakwaa et al., 2018). Feature selection was performed using the “randomForestSRC” package. MDS and NMDS were preferred to reduce the high dimensional data. Then, the importance of the candidate genes was ranked using the random forest of survival algorithm (nrep = 1000, which means the number of iterations of the Monte Carlo simulation is 1000), and the genes with relative importance greater than 0.3 were identified as key genes. The top 30 genes ranked by importance were used for mapping.

Second, feature genes are screened using the Least Absolute Shrinkage and Selection Operator (LASSO), a machine learning algorithm that screens out important variables and constructs optimal classification models by applying an L1 penalty (lambda) to set the coefficients of less important variables to zero. The degree of LASSO regression complexity adjustment is controlled by the parameter λ. The larger the value of λ, the greater the penalty for a linear model with more variables, resulting in a final result with fewer variables and more representative key genes (Zhang et al., 2021). To determine the optimal value of λ, we then performed 10-fold cross-validation and screened the most critical central genes by passing the selected λ. LASSO analysis was performed using the glmnet package.

Third, feature genes were screened using Support Vector Machine-Recursive Feature Elimination (SVM). SVM analysis is a widely used supervised machine learning technique to identify the best core genes by removing the feature vectors generated by SVM. We ran the SVM algorithm using the “e1071”software package to identify feature genes through a 5-fold cross-validation model.

## 3 Results

### 3.1 Acquisition of data from single-cell sequencing

**FIGURE 2:**
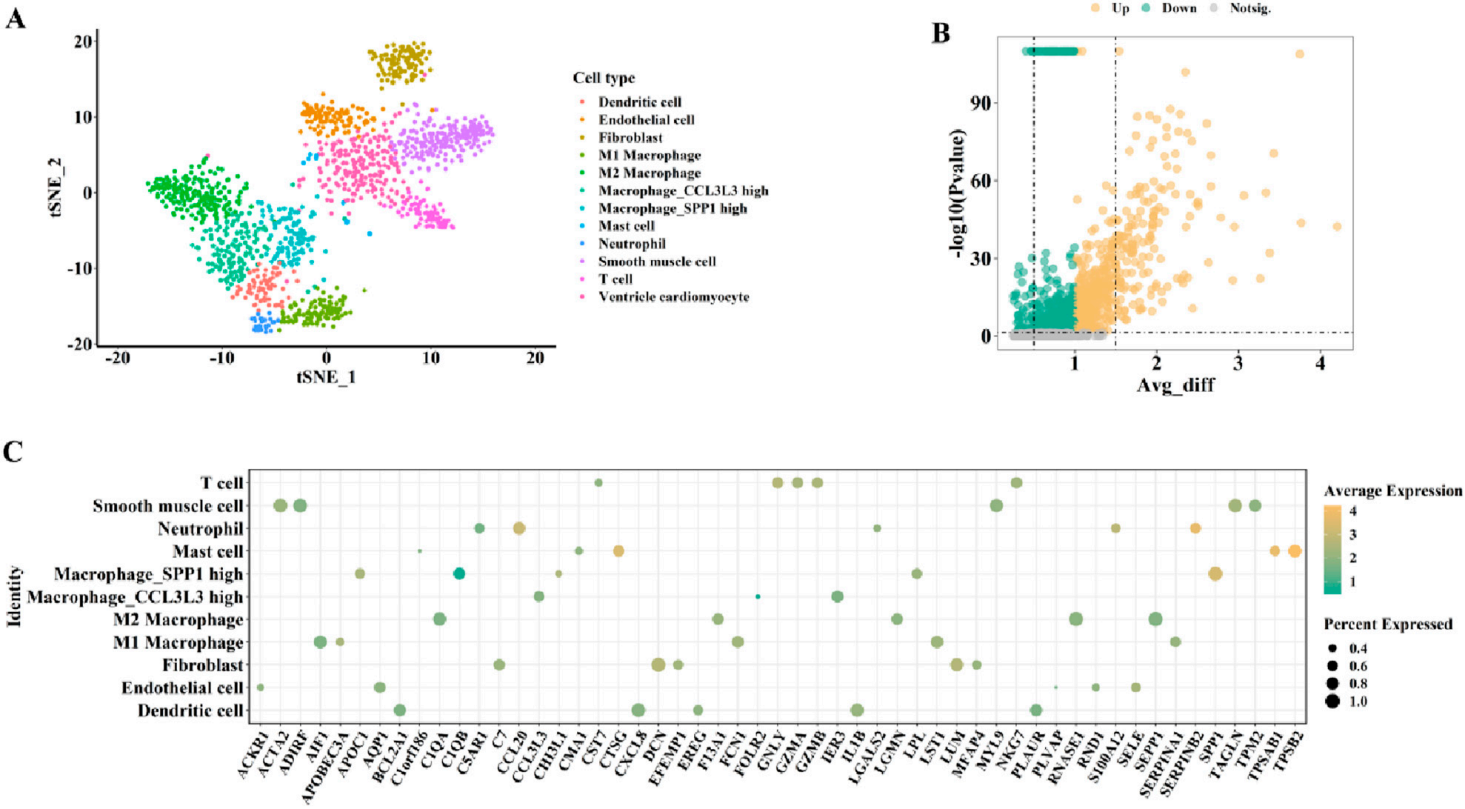
The cardiac-related gene markers (SCS data) for male from HCL database. **(A)** The SCS data was divided into 12 cell clusters. **(B)** The expression differences of all cardiac-related genes from HCL database (volcano diagram). **(C)** Expression trends of the top 50 candidate gene markers in different cell clusters.

**FIGURE 3:**
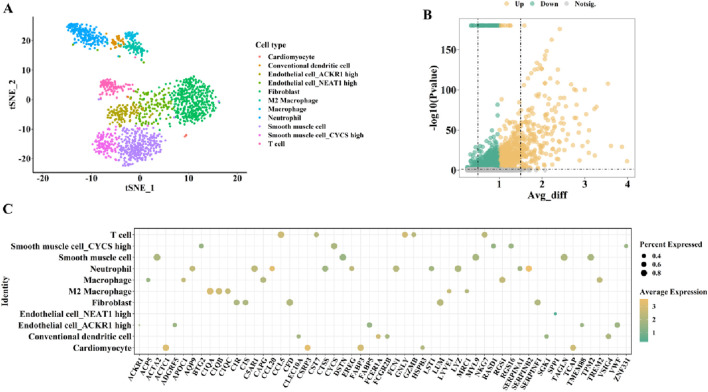
The cardiac-related gene markers (SCS data) for female from the HCL database. **(A)** The SCS data was divided into 11 cell clusters. **(B)** The expression differences of all cardiac-related genes from HCL database (volcano diagram). **(C)** Expression trends of the top 50 candidate gene markers in different cell clusters.

**FIGURE 4:**
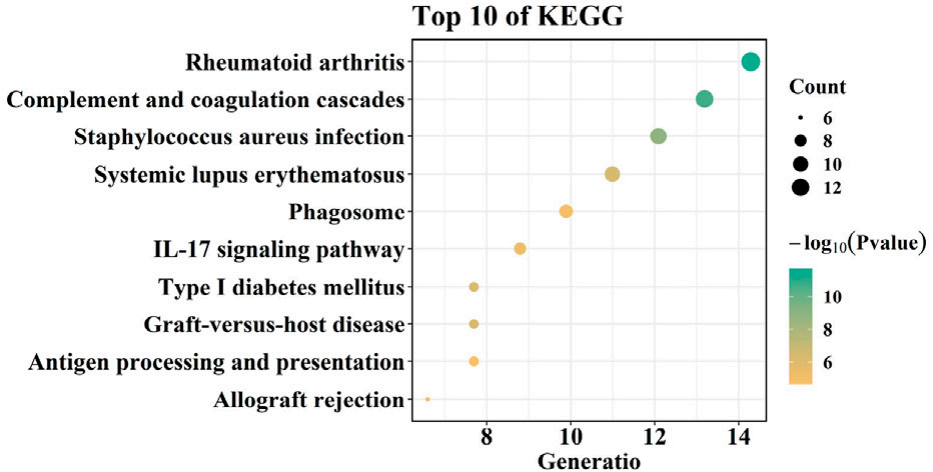
KEGG analysis of cardiac-related genes defined from SCS data.

To comprehensively identify key genes potentially associated with the transition from AS to HF in cardiac tissues, we first performed a preliminary screening of differentially expressed genes (DEGs) across various cell subpopulations using cardiac single-cell transcriptomic data from the Human Cell Landscape (HCL) constructed by Han et al. The SCS data were obtained from the HCL database and plotted (Figure 2 for males and Figure 3 for females). First, the t-distributed stochastic neighbor embedding (t-SNE) algorithm was used to nonlinearly dimensionalize the high-dimensional data, and we successfully classified 12 cell clusters (male, Figure 2A) and 11 cell clusters (female, Figure 3A). We then identified candidate gene markers in each cell cluster with screening thresholds for candidate gene markers: p < 0.05 (Figures 2B, 3B). Figures 2C, 3C show the expression trends of the top 50 candidate gene markers in different cell clusters. The larger diameter and more orange-red color of the circle represents the more significant expression of the gene. We organized the SCS data for males and females separately to obtain the candidate gene markers in the two data sets. If there were duplicated genes between two data sets, the genes in the same cell type were selected as overlapping genes. Based on the SCS data, we obtained a total of 2828 heart-related genes (1266 for adult heart 1, 1562 for adult heart 2), and in addition, we analyzed these genes for KEGG enrichment, and the results are shown in Figure 4. Through t-SNE dimensionality reduction and clustering of cardiac samples, we successfully identified multiple cell populations and extracted significantly expressed candidate biomarkers, providing high-resolution, tissue-specific gene resources for subsequent association analyses.

### 3.2 Identification of gene modules strongly associated with AS

**FIGURE 5:**
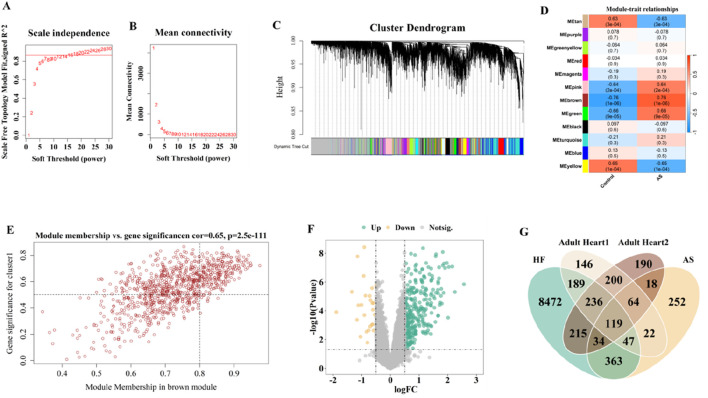
Selection of gene modules associated with AS though WGCNA and DEGs associated with HF by limma. **(A,B)** Soft thresholding power (β) selection via scale independence and average connectivity. **(C)** Gene modules associated with AS were shown in different colors below the cluster dendrogram. **(D)** The heatmap displays the |GS| values for modules of different colors. The larger the |GS| of a module, the stronger it is suggested to be associated with AS. **(E)** The correlation of MEbrown and AS. **(F)** The volcano map depicted the DEGs in HF. **(G)** The venn identified the overlaping genes from cardiac-related genes, genes associated with AS, and DEGs in HF.

To identify functional modules highly associated with AS, we performed WGCNA on the GSE28829 dataset. Compared with traditional differential expression analysis, WGCNA better captures the regulatory relationships among genes under complex disease conditions, making it more suitable for uncovering key modules tightly linked to the disease-related network structure. Gene modules strongly associated with AS were identified by WGCNA analysis of the GSE28829 dataset. During the construction of co-expression network, the most acceptable soft threshold β = 16 was chosen based on scale independence and average connectivity (Figures 5A,B).

Average association hierarchical clustering was performed based on TOM dissimilarity, and genes with similar expression profiles were classified into the same module, and a total of 12 co-expression modules of genes related to AS were obtained, which were indicated by different colors (Figures 5C,D). Figure 5D shows the GS_i_ values of different modules, and the modules with |GS_i_|>0.5 were considered as key gene modules in this study. We found five color modules, including MEtan, MEpink, MEbrown, MEgreen, and MEyellow, all of which had |GS_i_| > 0.5; however, the |GS_i_| of MEbrown module was the largest, suggesting that MEbrown might be a key gene module (918 genes; cor = 0.65; P = 2.5e- 111). Therefore, based on the correlation coefficients and *P* values, we selected MEbrown as the key gene module for the next analysis (Figures 5D,E).

### 3.3 Selection of overlapping genes associated with HF progression

The limma package was utilized to identify 9675 DEGs from the GSE57345 dataset, of which 4446 were upregulated and 5229 were downregulated (Figure 5F). To identify key driver genes involved in the transition from AS to HF, we integrated heart-expressed genes filtered from single-cell transcriptomic data, AS-associated hub genes identified by WGCNA, and HF-related DEGs from the GSE57345 dataset. Candidate overlapping genes were selected based on their intersection, ensuring strong biological relevance. This strategy guarantees that the selected genes possess cell-type specificity, disease association, and significant expression changes, thereby enhancing the scientific rigor and representativeness of subsequent modeling. Subsequently, 119 overlapping genes were selected from the cardiac-related 2828 genes screened by SCS data, 918 genes associated with AS identified by WGCNA, and 9675 DEGs associated with HF detected by the limma package (Figure 5G).

### 3.4 Functional enrichment analysis and PPI network construction

**FIGURE 6:**
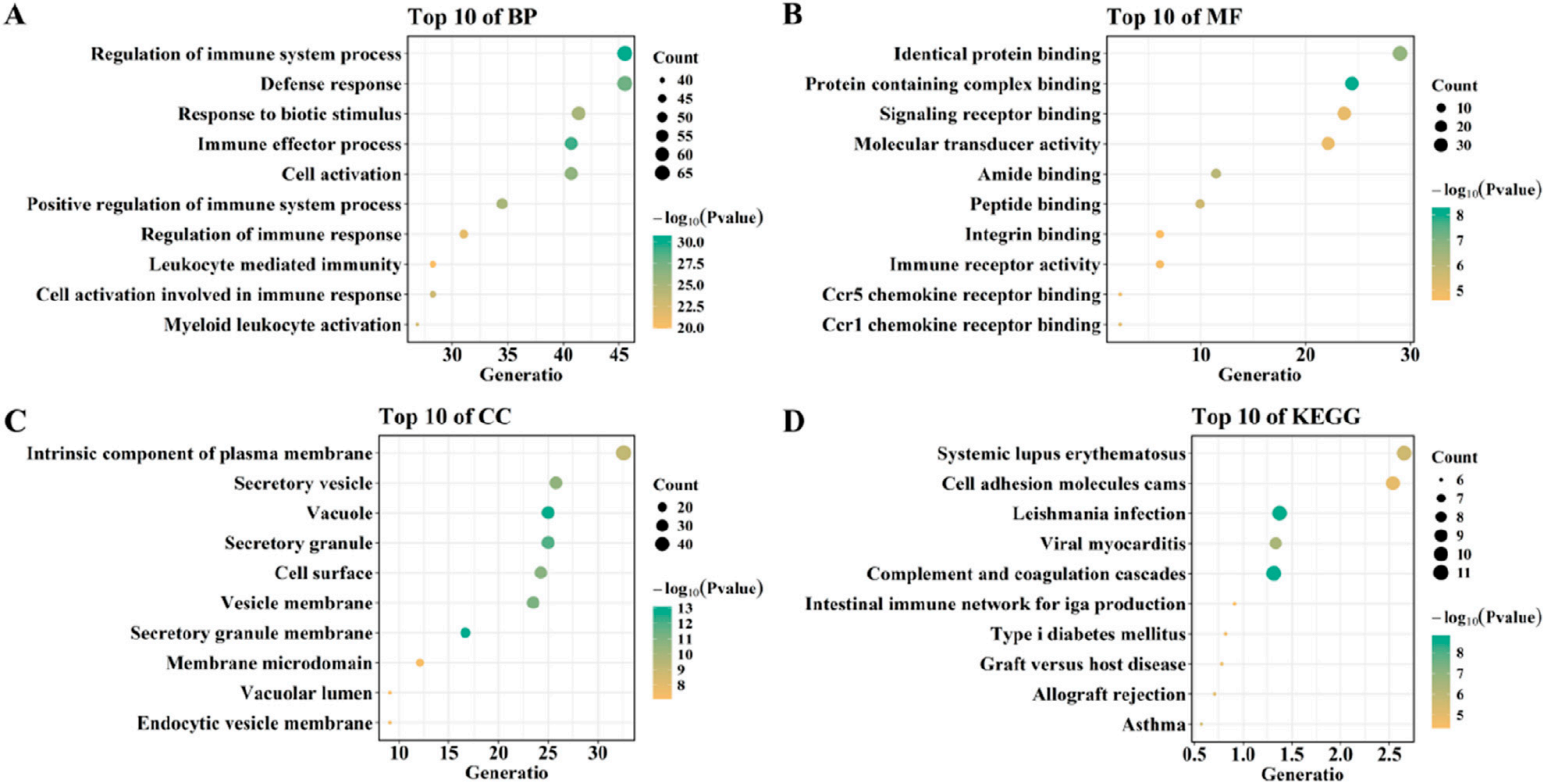
Functional enrichment analysis of overlaping genes. **(A–C)** GO analysis of overlaping genes. **(D)** KEGG analysis of overlaping genes.

To explore the functions of overlapping genes, GO, KEGG, and DO enrichment analyses were carried out using the “clusterProfiler” package and the “DOSE” package. GO analysis showed that biological processes included Regulation_of_immune_system_process, Immune_effector_process, Defense_response, Cell_activation, and Response_to_biotic_stimulus. Molecular functions include Protein containing complex binding, Identical protein binding, Amide binding, Peptide binding, and Ccr1 chemokine receptor binding. Cellular components included Secretory granule membrane, Vacuole, Secretory granule, Vesicle membrane, Cell surface (Figures 6A–C).

By using QuickGO, these terms have the same GO term (GO:0002376; immune system process) was selected depend on Ancestor Chart. Next, we analyzed the key genes in two datasets (GSE28829 for AS and GSE57345 for HF) with three machine learning methods (RF, LASSO, and SVM) after overlapping them with this GO term (GO:0002376; immune system process) and compared the differences key genes in GSE28829 were analyzed with three machine learning methods (RF, LASSO, and SVM) after overlapping them with this GO term (GO:0002376; immune system process) and compared the differences. 0002376; immune system process) after overlapping, the common genes screened by the three machine learning were GRB2, TNFSF13, LGALS9, SASH3, PAFAH1B1, LSM14A, CCL7, OPTN, and CCL4 (Supplementary Table S1); the key genes in GSE57345 genes overlapped with GO term (GO:0002376; immune system process), the common genes identified by the three machine learning screens were VSIG4, FCER1G, IFIT3, IFI44L, CCL5, CTSK, MCOLN1, HIF1A, CXCL12, GBP5, CD14, OAS1, JAM3, HLA.DPB1, CD48, PRKCD, DYSF, NAGK, KCNQ1, ORAI1, and IFITM1 (Supplementary Table S2).

**FIGURE 7:**
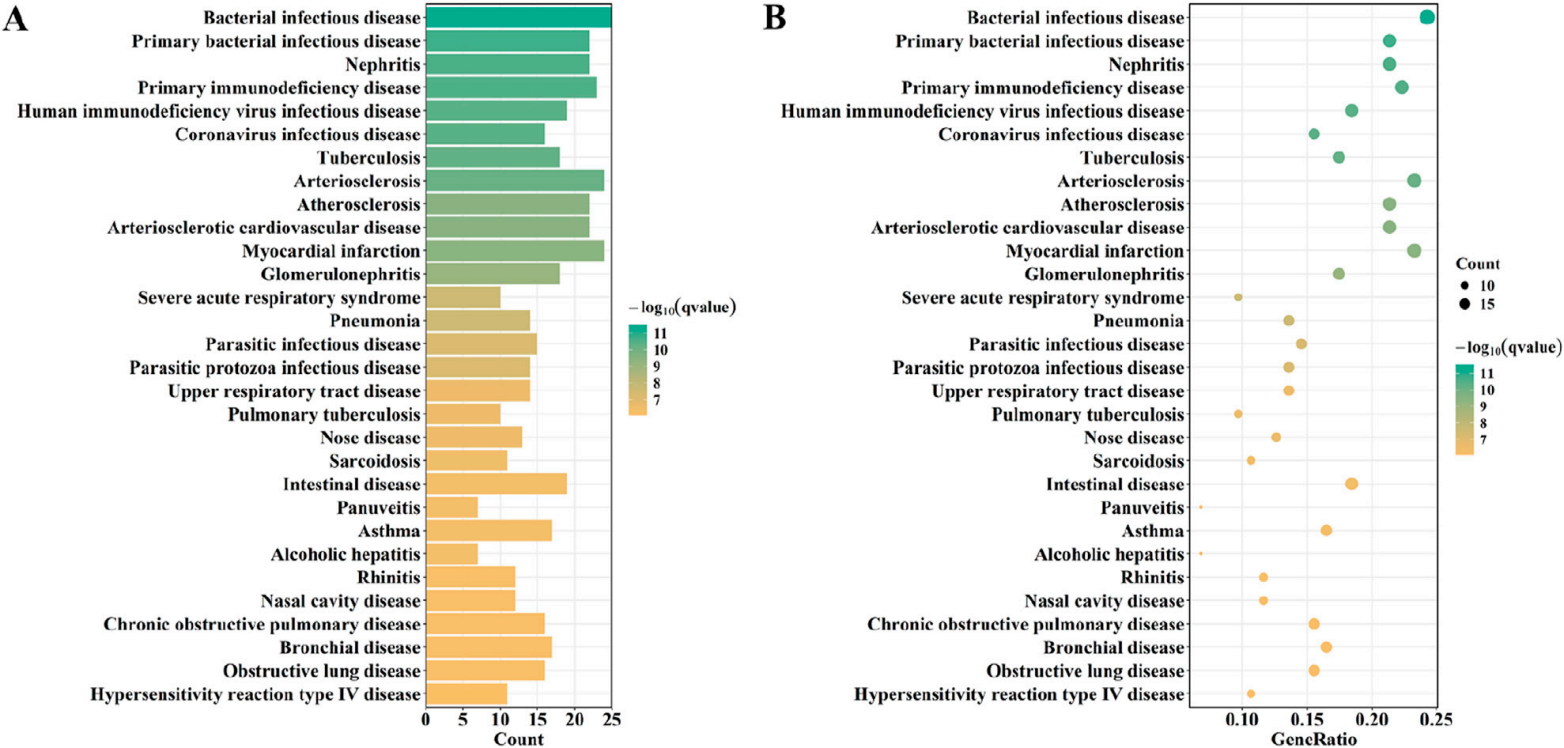
Do analysis of overlaping genes.

KEGG analysis showed that overlapping genes were involved in Complement and coagulation cascades, Viral myocarditis, Systemic lupus erythematosus, and, Asthma (Figure 6D). DO enrichment analysis showed that the expression of overlapping genes was associated with the development of bacterial infectious disease, primary bacterial infectious disease, nephritis, primary immunodeficiency disease, and human immunodeficiency virus infectious disease (Figure 7).

**FIGURE 8:**
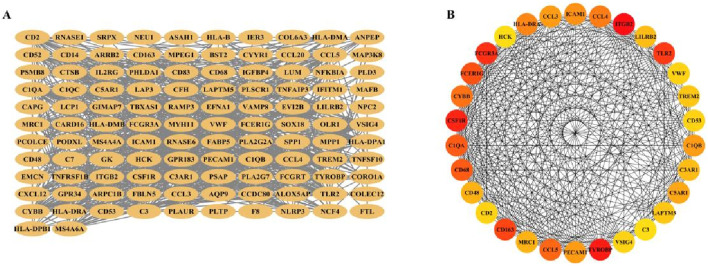
PPI network construction and node gene selection. **(A)** The PPI network showed the total interactions of 119 overlaping genes. **(B)** The top 30 node genes were identified based on the CytoHubba module of the Cytoscape software.

Next, 119 overlapping genes were analyzed for PPI by String, a PPI network with 118 gene nodes and 914 edges was constructed (Figure 8A). The 119 overlapping genes were then analyzed using the CytoHubba module of Cytoscape software, and the top 30 gene interactions were obtained based on the degree (Figure 8B).

### 3.5 Screening biomarkers using machine learning strategies and external validation

To further identify representative candidate biomarkers from the 119 overlapping genes, we employed three machine learning algorithms—RF, LASSO, and SVM—to perform feature gene selection, thereby enhancing the stability of feature selection through cross-validation. First, the 119 overlapping genes were fed into three different machine learning models (RF, LASSO, and SVM) for analysis to obtain the feature genes in GSE28829 dataset.

**FIGURE 9:**
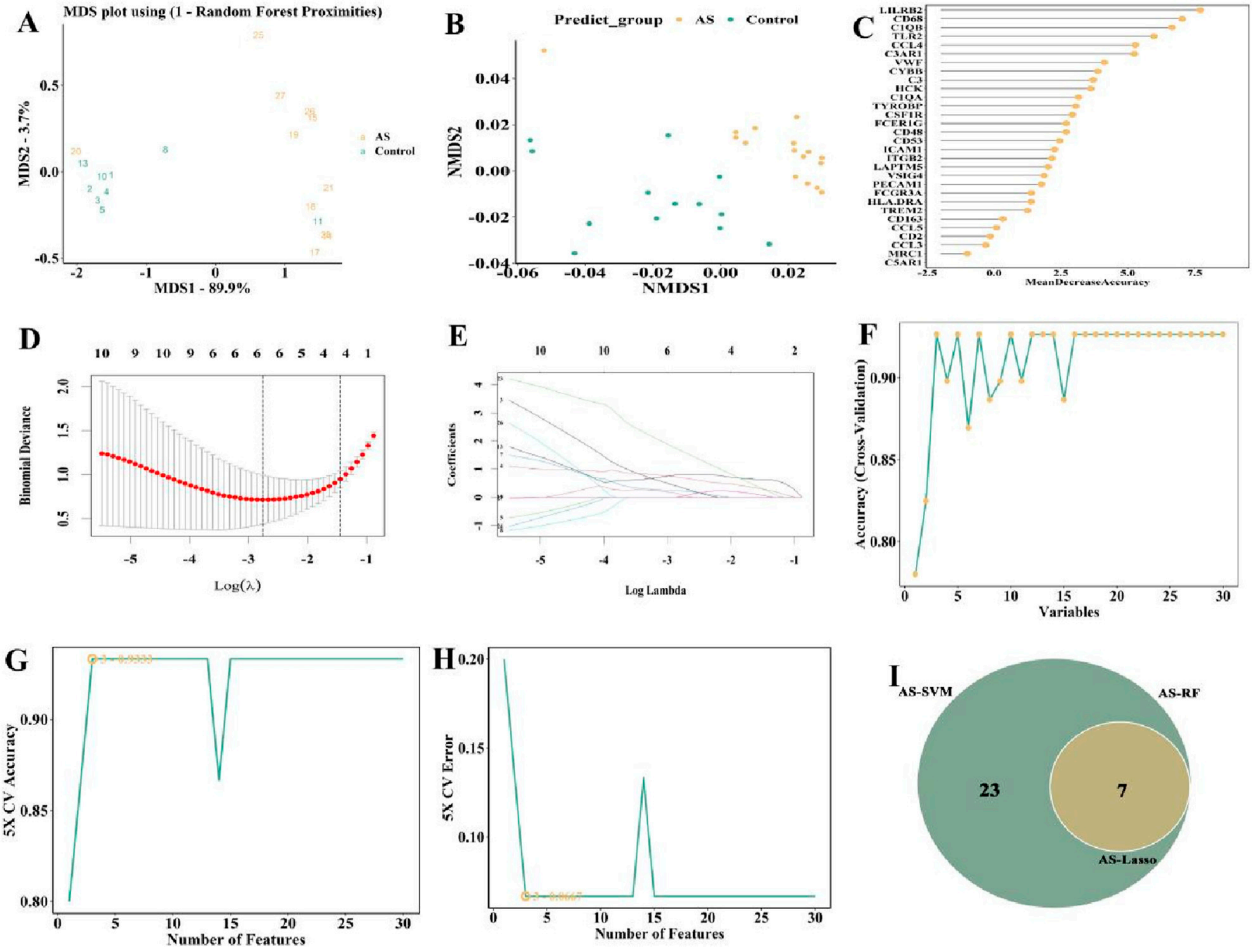
Selection of candidate biomarkers of in GSE28829 (AS patients) with RF, LASSO and SVM approaches. **(A)** MDS and **(B)** NMDS dimensionality reduction. **(C)** Ranking of gene importance score. **(D)** Determination the optimal value of λ and **(E)** Lambda.min value and a lambda.1se value. **(F–H)** SVM was applied to screen biomarkers based on the top 30 node genes. **(I)** The intersection of three machine learning algorithms was obtained with a venn.

The “randomForest” package was used to run RF to model the 119 overlapping. After MDS and NMDS dimensionality reduction, the random forest model was able to accurately identify the feature genes (Figures 9A,B). The 119 overlapping genes were then ranked according to the gene importance score (Figure 9C), and we took the top 30 genes (Supplementary Table S3) as candidate genes for the next round of analysis.

The characterization genes were also identified using LASSO regression. The 119 overlapping genes were entered into the LASSO regression with a lambda.min value of 0.063 and a lambda.1se value of 0.236. The results of LASSO regression showed that among the 119 overlapping genes, 7 genes (Supplementary Table S3) had the least binomial bias (Figures 9D,E), so these 7 genes were included in the next analysis.

The SVM algorithm was run using the “e1071”package. Through 5-fold cross-validation, the SVM method identified top thirty genes (Supplementary Table S3), indicating that these top thirty genes had the lowest error in detecting the AS and the highest accuracy after 100-fold cross-validation (Figures 9F–H), and were selected to be included in the subsequent analysis.

The candidate genes from GSE28829 were detected by the three methods (RF, n = 30; LASSO, n = 23; SVM, n = 30) were visualized as Venn plot (Figure 9I).

**FIGURE 10:**
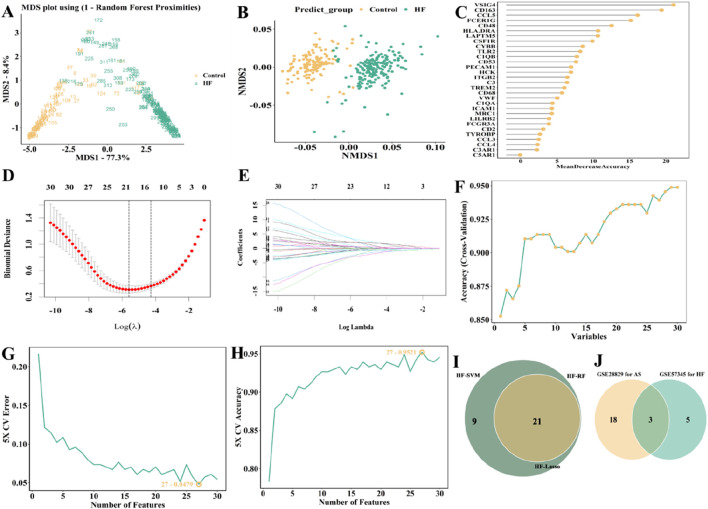
Selection of candidate biomarkers of in GSE57345 (HF patients) with RF, LASSO and SVM approaches. **(A)** MDS and **(B)** NMDS dimensionality reduction. **(C)** Ranking of gene importance score. **(D)** Determination the optimal value of λ and **(E)** Lambda.min value and a lambda.1se value. **(F–H)** SVM was applied to screen biomarkers based on the top 30 node genes. **(I)** The intersection of three machine learning algorithms was obtained with a venn. **(J)** The AUC obtained from ROC of two genes.

Subsequently, we also used the same method above to acquire the feature genes from GSE57345. The candidate genes were visualized as Venn plot as well (Figures 10A–I; Supplementary Table S4). Finally, C3, CCL4, and CD48 were identified as three overlapping genes for GSE28829 and GSE57345 (Figure 10J).

**FIGURE 11:**
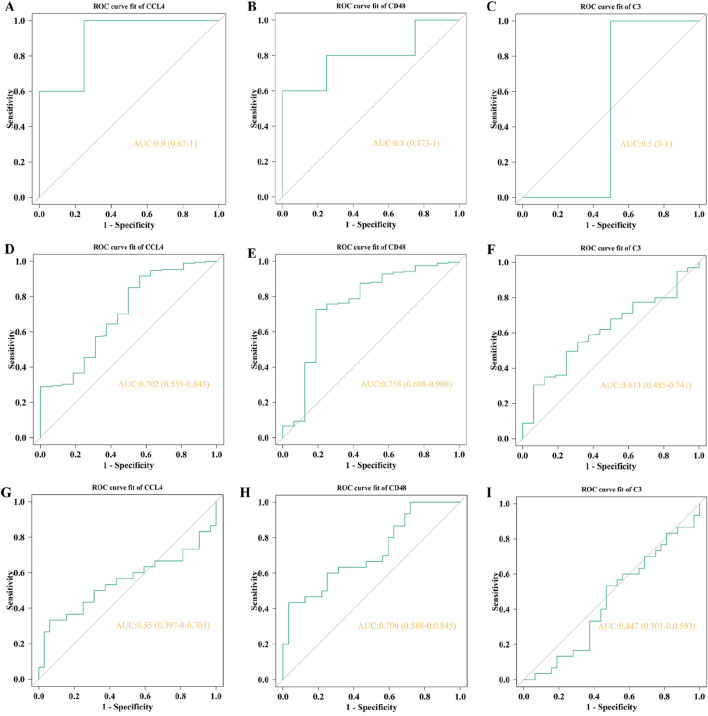
The AUC of external validation. The AUCs of CLL4 **(A, D, G)**, CD48 **(B, E, H)**, and C3 **(C, F, I)** in GSE53274, GSE5406, and GSE59867, respectively.

For the external validation, the GSE53274 dataset exhibits AUCs of 0.9 for CCL4 and 0.8 for CD48, as illustrated in Figures 11A–C, both of them were higher than 0.75. The AUCs of CD48 in the GSE5406 dataset and GSE59867 dataset are 0.758 and 0.706, respectively (Figures 11D–F; Figures 11G–I). CD48 demonstrated stable and relatively high predictive performance across multiple datasets (AUC >0.75), indicating its potential as a robust biomarker with broad applicability and clinical translational value in the progression from AS to heart failure HF.

## 4 Discussion

AS is a slowly progressive form of aortitis and is a leading cause of death worldwide (Fardoun et al., 2017).

Vascular embolism caused by advanced unstable plaque detachment increases the chance of HF and is highly life-threatening (Kong et al., 2022). AS is a multistep pathologic process involving multiple factors, and the search for specific key genes and pathways to predict its progression to heart failure is critical for early treatment of patients. Previous studies have explored pivotal genes associated with AS (Wen et al., 2023; Sun and Li, 2023), respectively; however, no study has explored the mechanisms underlying the progression of HF in patients with AS at the genetic level. To our knowledge, this is the first study to perform four rounds of biomarker screening using a comprehensive suite of computational methods to identify a candidate gene for early detection of HF progression in AS patients, aiming to provide novel insights for patient management.

Scholars now believe that the basis of atherosclerosis pathology progresses from lipid accumulation to local and systemic inflammation (Lu and Daugherty, 2015). Atherosclerosis is driven by lipid accumulation in the arterial wall, inflammation, and vascular damage. As the disease progresses, some plaques tend toward an unstable phenotype with more severe inflammation. Eventually, the plaque ruptures and an occlusive thrombus forms when blood comes into contact with the plaque contents, which induces myocardial infarction and ultimately leads to cardiomyocyte death, impaired cardiac function and heart failure. In this study, we identified a total of 119 overlapping genes in the two diseases, and GO and KEGG pathway enrichment analyses showed that these genes were significantly enriched in inflammatory and immune pathways, including Immune response, Inflammatory response, and Innate immune response, which are collectively involved in arterial AS and HF onset and progression. Cellular necrosis and macrophage infiltration have been shown to be key features of plaque vulnerability (Dohi et al., 2013; Hansson et al., 2015). Inhibiting macrophage proliferation and infiltration inhibits atherosclerotic plaque formation, reduces local inflammation and decreases the necrotic core area, thereby improving plaque stability and preventing progression to malignancy (Yamada et al., 2018; Chen et al., 2017).

In the last decade, science has made significant progress, especially with the rapid development of bioinformatics and machine learning strategies. Single-cell sequencing (SCS), a new technology for high-throughput sequencing of genomes, transcriptomes, and epigenomes at the single-cell level, plays an important role in the treatment of cardiovascular diseases. Secondly, limma analysis and WGCNA are used to screen gene clusters for connected and overlapping genes in AS and HF. Machine learning algorithms have a wide range of applications in biomedicine and have shown excellent efficiency in clinical trials (Zhou et al., 2022; Fu et al., 2018). However, few studies have integrated these several approaches to investigate the role of mRNAs in patients with atherosclerosis regarding the progression of HF. We integrated these analytical tools, including PPI network analysis and nomogram assessment, to identify a robust biomarker, CD48, for predicting HF progression in AS patients.

CD48, a member of the immunoglobulin superfamily (IgSF), belongs to the CD2 subfamily of signaling lymphocyte activation molecules (SLAMs). It is a glycosylphosphatidylinositol (GPI)-anchored protein predominantly expressed on the membrane surfaces of antigen-presenting cells and T cells, particularly lymphocytes, dendritic cells, and endothelial cells (McArdel et al., 2016). Functionally, CD48 plays a crucial role in the T cell activation cascade (Rudi et al., 2021). By interacting with its high-affinity ligand CD2—a key molecule in T cell activation—CD48 promotes T cell proliferation and facilitates intercellular communication between T cells and other immune cells (Muhammad et al., 2009). The CD48–CD2 interaction is pivotal in modulating the magnitude and quality of immune responses (Del Porto et al., 1991). Although no studies have yet definitively established a direct link between CD48 and the pathogenesis of atherosclerosis (AS), myocardial infarction (MI), or heart failure (HF), CD48’s role in immune regulation suggests its potential involvement. Chronic inflammation is widely recognized as a major driver of plaque destabilization in advanced atherosclerosis (Ley et al., 2011). Histopathological analyses of advanced human plaques reveal dense immune cell infiltration, with T cells accounting for approximately 50%–65% of infiltrating cells (Depuydt et al., 2020). These T cell subsets secrete pro-inflammatory cytokines that contribute to plaque vulnerability and rupture. Additionally, senescent T cells have been shown to accumulate intracellular cholesterol, further promoting vascular inflammation and instability (Bazioti et al., 2023). A recent bioinformatics study identified CD48 as a candidate diagnostic biomarker for acute myocardial infarction (AMI), highlighting its potential utility in early diagnosis and risk stratification (Jin et al., 2024). In this study, we observed that CD48 exhibited stable expression patterns in both diseased tissues and peripheral blood samples, suggesting that CD48 may be involved in a common pathway linking aortic stenosis (AS) to heart failure (HF) progression. However, further experimental validation is required to confirm this association. Notably, CD48 exists in two forms: membrane-bound (mCD48) and soluble (sCD48) (McArdel et al., 2016). The soluble form can be quantitatively measured in plasma or serum. Gangwar et al. reported significantly elevated sCD48 levels in patients with mild asthma and proposed a threshold of >1482 pg/mL as the optimal diagnostic cutoff to distinguish asthma patients from healthy controls (G et al., 2017). These findings indicate that sCD48 is detectably expressed in peripheral blood, highlighting its potential as a feasible non-invasive biomarker. Given the stable expression of sCD48 in peripheral blood and its close association with immune responses, it holds promise as an early warning biomarker for HF progression in AS patients. sCD48 could be integrated into a multimodal diagnostic framework that combines enzyme-linked immunosorbent assay (ELISA)-based non-invasive sCD48 detection with conventional imaging modalities—such as echocardiography for left ventricular ejection fraction (LVEF) assessment—and biochemical markers, including N-terminal pro–B-type natriuretic peptide (NT-proBNP), to enhance risk stratification and disease monitoring.

Our Gene Ontology (GO) and Kyoto Encyclopedia of Genes and Genomes (KEGG) enrichment analyses revealed that differentially expressed genes were primarily enriched in inflammatory responses, T cell receptor signaling pathways, and other related processes, which play pivotal roles in the progression from atherosclerosis to heart failure. Previous studies have demonstrated that the rupture of atherosclerotic plaques and subsequent myocardial remodeling are characterized by chronic inflammation mediated by T lymphocytes and concomitant reactive fibrosis (Anzai, 2018). Chronic inflammatory states accelerate heart failure development by promoting cardiomyocyte apoptosis, collagen deposition, and ventricular remodeling. Thus, our enrichment results provide multi-omics level support for the critical role of immune-inflammatory networks in AS-to-HF transition.

Using the HCL database, we demonstrated significant differences in cardiac cell clusters between male and female subjects based on SCS data. The t-SNE clustering further indicated sex-specific variations in the composition of immune cells and cardiomyocyte subtypes, suggesting potential sex differences in the cardiovascular immune microenvironment. Studies have reported that sex influences immune regulation mechanisms in AS and HF. Female AS patients typically exhibit stronger humoral immune responses (Fairweather, 2015), whereas males show elevated T cell–mediated inflammatory activity (Klein and Flanagan, 2016). This sex-specific immune response pattern may indirectly affect CD48-mediated T cell activation and immune-inflammatory signaling pathways. Moreover, the function and phenotype of these immune cells are modulated by sex hormones such as estrogen and testosterone (Trigunaite et al., 2015), potentially leading to differential effects on CD48 expression and function between sexes and further shaping the immunopathological characteristics during AS-to-HF progression.

The results of this study could have deeper applications in both basic and clinical research. For basic research: first, there is a need to validate the expression of the identified genes in a combined model of AS and HF. For example, a combined model of AS and HF can be established by performing coronary artery ligation in ApoE^−/−^ mice. Later, at unused time points, heart tissue and peripheral blood are collected and tested for expression of the two candidate genes. Second, adeno-associated virus (AAV) was injected into the mice to alter gene expression, and ultrasound, electrocardiography, and biochemical tests were performed to assess whether the gene expression changes affected the progression of HF in the atherosclerotic mice; third, high-throughput sequencing was performed on cardiac tissues and peripheral blood to gain a deeper understanding of the intrinsic mechanisms of HF progression. These experiments have not been performed in this present study, and this is a research idea that we would like to conduct in the future. Based on the above research tools, we hope to identify and validate biomarkers of HF progression in atherosclerotic patients, which will facilitate the implementation of early interventions for atherosclerotic patients to avoid health deterioration due to plaque rupture. For clinical research: After clarifying the biomarkers of HF progression in atherosclerotic patients and their intrinsic mechanisms, clinical studies can be designed at a later stage to improve the application value. For example, patients with AS are recruited, and based on the results of imaging and biochemical tests, etc., participants are categorized into two groups (low or high), and then samples are collected to verify the results.

Although our study provided novel insights, there were some limitations. First, the markers identified in this study were not fully consistent with the results of the external validation, and we speculate that this may be due to the following reasons: first, in the analysis of transcriptome datasets, the number of genes is often much larger than the number of samples, which may lead to overfitting. Certainly, to minimize the risk of overfitting in this study, we applied k-fold cross-validation during model training (10-fold cross-validation for LASSO; 5-fold cross-validation for SVM), while the Random Forest (RF) model was subjected to 1,000 iterations of Monte Carlo simulations to enhance result robustness; Second, different datasets may have used different sequencing platforms and there are differences in data cleaning and normalisation methods, and these methodological differences may also lead to inconsistencies in the efficacy of markers across datasets; Third, the population characteristics of the external validation dataset are not exactly the same as the test dataset, e.g., race, age, baseline disease status, Fourth, because GSE59867 is a whole blood dataset, which differs from other datasets in terms of tissue type, gene efficacy in blood samples may not be as high as in plaques and cardiac tissue, which may be related to disease progression. In future studies, we will provide more fine-grained control over data acquisition and processing, feature selection and model construction, and combine clinical sample testing with biological experiments to ensure the robustness of the markers.

Second, the differential gene screening threshold for single-cell sequencing was set at P < 0.05, a more lenient threshold, which may have implications for the results. On the one hand, the more relaxed threshold may lead to an increase in false positives. Screened genes with significant effect in the test set did not show effect in the external validation. On the other hand, too many candidate genes may lead to “clutter” in the results of functional enrichment analyses. We used a relatively loose threshold in the initial screening to capture as many candidate genes as possible, and then performed a second screening using multiple analysis of variance methods, retaining only those genes that performed stably across methods.

Third, because specific plaque tissues had to be used, the sample size was small. Since only one microarray dataset was analyzed for one disease and there was a lack of clinical information, this inevitably led to bias and population bias. This study combined analyses of both tissue and peripheral blood samples, thereby enhancing the translational potential of our findings. However, significant differences may exist between samples from different sources in terms of gene expression profiles, cellular composition, and the pathological states they reflect. For instance, peripheral blood samples predominantly represent systemic inflammatory status, offering advantages in clinical accessibility and dynamic monitoring, whereas tissue samples more directly capture pathological and biological changes within local lesions. Given that CD48 is expressed across various immune cell types, its expression changes in peripheral blood may be influenced by systemic immune activation, which could partially explain the somewhat lower predictive performance observed in blood samples. In contrast, CD48 expression in arterial or myocardial tissues is more likely to be closely associated with the local immune microenvironment. Future studies employing spatial transcriptomics or single-cell sequencing could further elucidate the precise expression patterns and functional roles of CD48 within specific cellular subpopulations and tissue microenvironments, thereby enhancing the specificity and clinical translational value of this biomarker.

Fourth, we applied only three different machine learning algorithms to cross-select the identified genes. When analysing transcriptome datasets, there are situations where the number of genes is much higher than the number of samples, which can lead to overfitting. Due to overfitting, the results of the internal test set may be better, but the results of the external dataset validation are poor. Therefore, we used LASSO, RF, and SVM to screen the genes. Overfitting effect due to small sample size and high data dimensionality in this study. When analysing transcriptome datasets, it is common to have a much larger number of genes than the number of samples, which can lead to overfitting. Due to overfitting, the results of the internal test set may be better, but the results of the external dataset validation are not satisfactory. Therefore, we used LASSO, RF and SVM methods to screen genes. Each algorithm has its own advantages, e.g., LASSO regression avoids overfitting (Li and Sillanpää, 2012); SVM-RFE is able to retain variables relevant to the outcome in datasets with fewer samples (Huang et al., 2014). RF algorithm is good at managing high-dimensional data and building predictive models (Blanchet et al., 2020). However, in fact, there are many other methods, including arithmetic mean, geometric mean and median, which have specific advantages in feature selection (Li et al., 2022). More algorithms (such as Elastic-Net, XGBoost, etc.) and integrated approaches should be jointly used to identify feature genes in subsequent studies, and external validation cohorts will be expanded to reduce the risk of overfitting and enhance the stability of the model. We systematically detailed the key parameters and cross-validation strategies of each machine learning algorithm in the Methods section to enhance result reliability and minimize the risk of overfitting.

Fifth, although we identified one biomarker for detecting the progression of HF in patients with AS using the dataset, further *in vitro* and *in vivo* experiments are necessary in the future to elucidate the regulatory mechanisms of CD48. Sixth, AS has both genetic and autoimmune etiologies, and its pathogenesis needs to be further explored based on etiology.

In conclusion, this study has identified a potential biomarker, CD48, using bioinformatics and machine learning algorithms, which provides a model for detecting atherosclerosis patients who have progressed to heart failure and also suggests potential therapeutic targets. In summary, we anticipate that CD48 may serve as a clinically relevant biomarker for predicting AS-to-HF progression, enabling earlier intervention and potentially improving long-term outcomes for patients with atherosclerosis.

## Data Availability

The datasets presented in this study can be found in online repositories. The names of the repository/repositories and accession number(s) can be found in the article/Supplementary Material.

## Abbreviations

AS, Atherosclerosis; HF, Heart failure; SCS, Single-cell sequencing; HCL, Landscape online platform; GEO, Gene expression omnibus; DEGs, Differentially expressed genes; WGCNA, Weighted gene co-expression network analysis; AS, Atherosclerosis; HF, heart failure; GO, Gene ontology analysis; KEGG, Kyoto Encyclopedia of the Genome; DO, Disease ontology; LASSO, Least Absolute Shrinkage and Selection Operator; SVM-RFE, Support Vector Machine-Recursive Feature Elimination; RF, Random Forest; ROC, Receiver Operating Characteristic; AUC, Area under the curve; apoB, Apolipoprotein B; CAD, Coronary artery disease; TOM, Topological overlap matrix; ME, Module eigengenes.
